# Cloning and expression of L-asparaginase from *Bacillus tequilensis* PV9W and therapeutic efficacy of Solid Lipid Particle formulations against cancer

**DOI:** 10.1038/s41598-018-36161-1

**Published:** 2018-12-20

**Authors:** Ganeshan Shakambari, Rai Sameer Kumar, Balasubramaniem Ashokkumar, Venkatachalam Ganesh, Vairathevar Sivasamy Vasantha, Perumal Varalakshmi

**Affiliations:** 1 Department of Molecular Microbiology, School of Biotechnology, Madurai Kamaraj University, Madurai, Tamil Nadu 625021 India; 2 Department of Genetic Engineering, School of Biotechnology, Madurai Kamaraj University, Madurai, Tamil Nadu 625021 India; 3 Electrodics and Electrocatalysis (EEC) Division, CSIR - Central Electrochemical Research Institute, (CSIR - CECRI), Karaikudi, Tamilnadu 630003 India; 4 Department of Natural Products Chemistry, School of Chemistry, Madurai Kamaraj University, Madurai, Tamil Nadu 625021 India

## Abstract

L-asparaginase, a therapeutic involved in cancer therapy, from *Bacillus tequilensis* PV9W (*ans*A gene) was cloned and over expressed in *Escherichia coli* BL21 (DE3), achieved the aim of maximizing the yield of the recombinant enzyme (6.02 ± 1.77 IU/mL) within 12 h. The native L-asparaginase of *B. tequilensis* PV9W was encapsulated using solid lipid particles by hot lipid emulsion method, which is reported for first time in this study. Subsequently, the lipid encapsulated L-asparaginase (LPE) was characterized by SEM, UV-Vis spectroscopy, FT-IR, SDS-PAGE and its thermo stability was also analyzed by TGA. Further characterization of LPE revealed that enzyme was highly stable for 25 days when stored at 25 °C, showed high pH (9) tolerance and longer trypsin half-life (120 min). In addition, the cytotoxic ability of LPE on HeLa cells was highly enhanced compared to the native L-asparaginase from *Bacillus tequilensis* PV9W. Moreover, better kinetic velocity and lower K_m_ values of LPE aided to detect L-asparagine in cell extracts by Differential Pulse Voltammetry (DPV) method. The LPE preparation also showed least immunogenic reaction when tested on normal macrophage cell lines. This LPE preparation might thus pave way for efficient drug delivery and enhancing the stability of L-asparaginase for its therapeutic applications.

## Introduction

L-asparaginase, an enzyme with applications in cancer therapy as well as food industry has been extensively researched for over four decades. Such a quest for a microbial source of the enzyme leads the isolation and characterization of many microorganisms from various niches. One such isolate *Bacillus tequilensis* PV9W is highlighted in the current study. Earlier studies with L-asparaginase from *Bacillus tequilensis* PV9W showed least K_*m*_ value when characterized for its substrate L-asparagine and its increased sensitivity to L-asparagine at lower concentration makes it an important enzyme from therapeutic point of view^1^.

Classical studies with the enzyme production by *Bacillus tequilensis* PV9W revealed the need of L-asparagine to induce the production and could not be substituted by cheaper substrates for cost effective production. In this study, an attempt was made to clone the genetic element responsible for L-asparaginase production in *Bacillus tequilensis* PV9W into another bacterial host and over-express the protein using recombinant DNA techniques.

Recombinant DNA technology plays an important role in enhancing production and properties of many commercially important enzymes like the L-asparaginase mentioned above, which has applications in therapeutics and food industry. Many attempts have been successfully made and implemented in cloning the gene responsible for L-asparaginase from organism like *Escherichia coli* where L-asparaginase is governed by different molecular elements in different organisms. *Escherichia coli* produce two L-asparaginases with markedly different properties, where L-asparaginase I, with a low affinity for L-asparagine, is cytoplasmic, produced constitutively while, L-asparaginase II is a high-affinity enzyme and is secreted into the periplasm, and its expression is positively regulated by inducers (cyclic AMP), receptor protein and anaerobiosis (FNR protein)^2^.

However, in *Bacillus* sp. the L-asparaginase is regulated by two different and independent pathway. First, the *ansZ* gene that encodes a functional L-asparaginase, where the expression is activated during nitrogen-limited growth by the TnrA transcription factor. Second, the *ans*A gene that is located in an operon along with *ans*B, which encodes L-aspartase and the expression of the *ans*AB operon is repressed by AnsR and the activity of AnsR has been proposed to be regulated by either asparagine or aspartate^3^. The cloning, over-expression, and characterization of the gene encoding L-asparaginase (*ans*Z) from a non-pathogenic strain of *Bacillus subtilis* B11−06 has been reported, the protein was successfully purified and characterized for its thermo-stability^4^. The PCR based screening of *ans*A gene in *Bacillus tequilensis* has been reported by Nayak *et al*.^5^. This study involves isolation of the *ans*A gene from *Bacillus tequilensis* PV9W and a first attempt in cloning of it in suitable strain for over expression in another bacterial host.

Furthermore, many different methods have been prospected to improve efficiency, drug delivery and storage of L-asparaginase by conjugating it to poly-ethylene glycol (PEG)^6^, to lipid conjugates by chemical bonding^7^ etc. However, the methods for such conjugation are complex and have many limitations. This work encompasses an attempt to synthesize solid lipid particles encapsulated L-asparaginase such that its efficacy is increased in cytotoxic applications and its correlation with L-asparagine depletion was proved using electrochemical methods.

## Results

### PCR amplification of *ans*A ORF from *Bacillus tequilensis* PV9W and TA cloning

The *ans*A gene was amplified from the genomic DNA of *Bacillus tequilensis* PV9W, which was visualized with a size of ~990 bp in the gel (Supplementary Fig. S1a). The sequence of the amplicon revealed to be 99% to L-asparaginase coding sequences (cds) from *Bacillus tequilensis* NIOS4 (Supplementary Fig. S1b). Moreover, the BLAST-n analysis of the sequence obtained belonged to conserved domains of L-asparaginase superfamily and the amino acid sequences thus derived from the nucleotide sequences could be seen to match with L-asparaginase from *Bacillus tequilensis* (Sequence ID: AFM80112.1) (Supplementary Fig. S1c).

The *ans*A was successfully ligated into pGEMT vector and transformed into *E. coli* DH5α. The plasmids from the clones containing the *ans*A insert were subjected restriction digestion with *EcoRI* and *HindIII* showed a release of 990 bp, which was sequenced and found to match with *Bacillus tequilensis* NIOS4 L-asparaginase cds, (Supplementary Fig. S2) and contained the complete ORF of L-asparaginase cds of *Bacillus tequilensis* which was translated by ExPASy Translate tool with methionine in the start and presence of stop codon. The translated amino acid sequence (329 excluding stop codon), was aligned with amino acid sequence from *Bacillus tequilensis strain* NIOS4 and showed 98% identity to L-asparaginase I (*Bacillus tequilensis*, GenBank: AFM80112.1).

### Sub cloning of *ans*A into pET 28a+ vector

The 990 bp *ans*A gene released by restriction digestion of pGEMT-*ans*A plasmid was ligated with the linearized pET28a+ plasmid, transformed into *E. coli* BL21 (DE3) and plated on LB plate containing kanamycin as selectable marker. The clones seen on kanamycin plate screened for the *ans*A insert and the colonies 1, 2 and 4 showed 990 bp gene amplified with primers specific to *ans*A. Further, restriction digestion of the plasmids obtained from clone 1 released a 990 base pair and the plasmid backbone and used for expression analysis (Supplementary Fig. S3). Further, the sequence of the *pET 28a-ans*A clone derived amplicon revealed identity with ansA gene from *Bacillus subtilis* (Supplementary Fig. S4). The sequence of *pET28a-ans*A clone amplicon contained the *ans*A ORF, and was translated into corresponding amino acid sequence using *ExPASy* (Supplementary Fig. S4). The sequence of *ans*A from *pET28a-ansA* clone was submitted to NCBI (GenBank acc no. *ans*A MF593111).

### Over expression of L-asparaginase by *pET28a-ansA* clone

**Figure 1:**
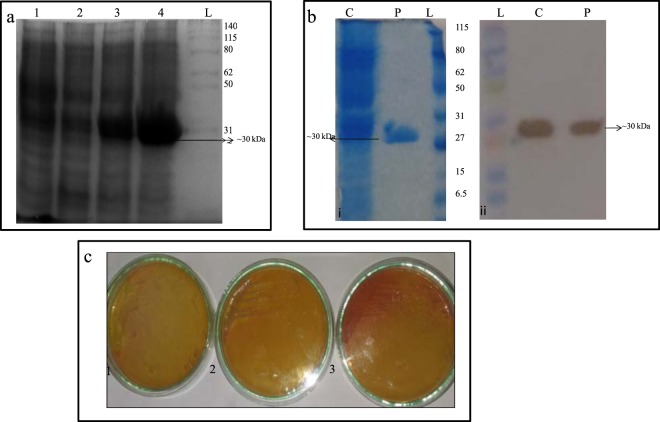
IPTG induction and analysis of recombinant L-asparaginase. (**a**) SDS-PAGE analysis of recombinant L-asparaginase produced by *pET 28a- ans*A (clone 1) performed for confirming IPTG induced over expression of L-asparaginase. L- Ladder; cell lysates of 1- *E. coli* BL21–control; 2- pET clone 1 uninduced; 3- *pET 28a- ans*A (clone 1) induced with 0.5 mM IPTG; 4- *pET 28a- ans*A (clone 1) induced with 1 mM IPTG. (**b**) Western Blot analysis of purified His-tagged L-asparaginase to confirm presence of the recombinant enzyme. (i) SDS-PAGE analysis of one half of gel, (ii) Second half gel was immunoblotted and the membrane treated with anti-His antibody was developed with DAB; L- Ladder; C- Crude cell lysate; P-Purified protein. (View Supplementary Plate 1–3) (**c**) L-asparaginase production screening on M-9 plate containing 1% L-asparagine and phenol red indicator by 1. *E. coli* BL21 –control; 2. *pET 28a- ans*A (clone 1)- un-induced; 3. *pET 28a- ans*A (clone 1)- induced with 1 mM IPTG.

Using different concentrations of IPTG (0.5 and 1 mM), over expression of a protein by *pET28a-ans*A clone was seen as a band at approximately 30 kDa size when the cell lysate was electrophoresed (SDS-PAGE) and this size is similar to the L-asparaginase produced by *Bacillus tequilensis* PV9W (Fig. 1a) and this band was not seen in the cell lysate of the control BL21 strain and un-induced *pET28a-ans*A clone. Further, 1 mM IPTG was used to induce L-asparaginase production in a 200 mL LB culture of *pET28a-ans*A clone and purified using Ni-NTA column to provide a quantitative yield of 6.02 ± 1.77 IU/mL enzyme activity. The purified protein of ~90 kDa and ~30 kDa was observed on native and SDS-PAGE respectively which was similar to native L-asparaginase (Supplementary Fig. S5a,b). The purification was efficient as no protein was lost in the column flow through or the washings as seen in the SDS and native PAGE analysis.

### Western blot analysis of the purified recombinant protein L-asparaginase produced by *pET28a- ans*A clone

The recombinant L-asparaginase produced by the *pET28a-ans*A clone was confirmed by western blot analysis using antibodies against the His tag of the protein produced. The results are depicted by Fig. 1b, where a 30 kDa band was seen in crude and purified samples of section of gel stained with Coomasie Blue and it was also substantiated when a section of the gel loaded with the cell lysate of *pET28a-ans*A clone and its purified product was electro blotted on nitrocellulose membrane and blotted for presence of poly his-tag using anti-His antibody of mouse origin. The blot developed by DAB reagent showed presence of His tagged protein at the same place as the purified protein (30 kDa) (Fig. 1b).

### Confirmation of production of functional recombinant protein L-asparaginase produced by *pET 28a- ans*A clone1

IPTG induced *pET28a-ans*A clone, un-induced *pET28a-ans*A clone and control *E. coli* BL21 was plated on M-9 plate with phenol red indicator and it was observed that *pET28a-ans*A clone 1 produced L-asparaginase capable of hydrolyzing L-asparagine and liberating ammonia thus causing a colour change to pink (Fig. 1c).

### Cytotoxic activity of recombinant protein L-asparaginase produced by *pET 28a- ans*A clone on HeLa cells

The recombinant L-asparaginase was found to be cytotoxic to HeLa cells (Supplementary Fig. S5c) as seen by microscopic observation after 12 and 24 h at IC_50_ = 0.044 ± 0.006 IU. This value is similar to that of the native L-asparaginase from *Bacillus tequilensis* PV9W (0.036 ± 0.009 IU) as reported earlier^1^.

### Bio compatible carrier particle for drug delivery- Solid Lipid Particle (SLP)

**Figure 2:**
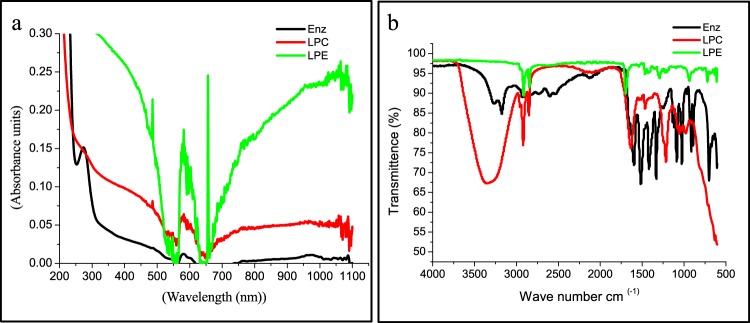
Spectroscopic analysis of LPE. (**a**) UV-Visible spectrum (**b**) FT-IR analysis of. L-asparaginase, Lipid particle-Control (no enzyme) and Lipid particle containing L-asparaginase.

In order to enhance the drug delivery and long lasting effect of the enzyme, the L-asparaginase from *Bacillus tequilensis* PV9W was encapsulated into solid lipid particles synthesized using palmitic acid. The confirmation and characterization of the encapsulated particles was done using spectroscopic methods wherein, UV-Visible spectroscopy, L-asparaginase showed a peak at 280 nm, empty lipid particles control (LPC) showed a small peak at nearly 600 nm, and the lipid particle containing L-asparaginase showed increased absorbance at 280 nm as well as at 600 nm additional peak at 650 nm (Fig. 2a). The spectrum clearly depicts efficient encapsulation of L-asparaginase in the lipid particle.

This fact was also confirmed by FT-IR spectral analysis (Fig. 2b) where L-asparaginase showed carboxyl functional group specific peaks between wave number 702 to 1589 cm^−1^, corresponding to a absorption peaks that account for a variety of single bonds namely C-H, C-O,O-H and C-S stretches. The LPC shows broad -OH peak at wave number 3356 cm^−1^ corresponding to polymeric O-H stretch and few peaks at 1635 cm^−1^ 1465 cm^−1^, 1218 cm^−1^, 1059 cm^−1^ and 978 cm^−1^. However, for lipid particle encapsulated L-asparaginase (Fig. 2b) sharp peaks at 2914, 2847 cm^−1^ and 1535 cm^−1^ similar to those of LPC and incorporation of peaks similar to L-asparaginase between 1351 to 721 cm^−1^ with slight chemical shifts from those of L-asp alone indicating encapsulation of the enzyme by the particle (Fig. 2b). Similar shifts were indicated as evidence of encapsulation of zeolitic imidazolate by curcumin for making an effective drug delivery system^8^ and in encapsulation of plasmid DNA by Chitosan-Saponin Nanoparticle^9^.

### Characterization of LPE

**Figure 3:**
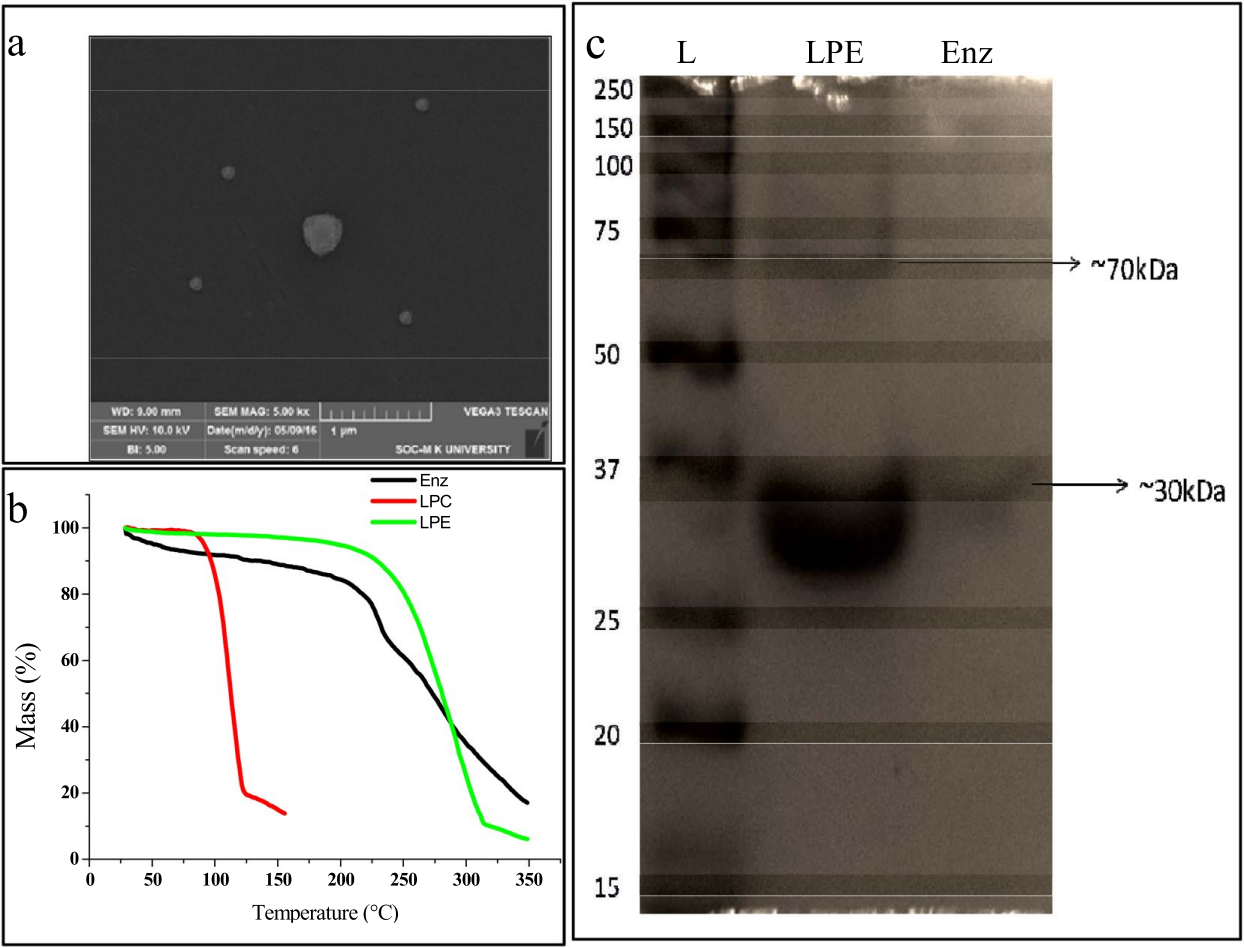
Characterization of LPE. (**a**) SEM analysis of lipid particle encapsulated L-asparaginase sputter coated with gold. (**b**) Thermogravimetric analysis of lipid particle encapsulated enzyme (LPE) and lipid particle control (LPC) L-asparaginase alone. (**c**) SDS PAGE analysis of L-asparaginase and lipid particle encapsulated L-asparaginase and purified L-asparaginase from *Bacillus tequilensis* PV9W (View Supplementary Plate 4).

Scanning Electron Microscopic (SEM) image (Fig. 3a) shows particles mostly in range of 100 nm. However some particles tend to be of bigger sizes too. This may be due to aggregation using poly vinyl alcohol (PVA).

The thermal stability of the lipid encapsulated enzyme (LPE) and L-asparaginase was revealed by thermogravimetric analysis studied as loss of mass (%) per unit increase in temperature and it was seen that LPE showed enhanced stability over a large range of temperature compared to the native enzyme from *Bacillus tequilensis* PV9W. Moreover, the empty control particles showed vulnerability to heat in the absence of L-asparaginase encapsulation (Fig. 3b).

It was interesting to note that the denaturation of the lipid encapsulated L-asparaginase brought about by SDS and mercaptoethanol in the lysis buffer used in SDS-PAGE analysis (Fig. 3c) caused the release of L-asparaginase showing a band of ~30 kDa, which corresponds to L-asparaginase from *Bacillus tequilensis* PV9W, while an additional band of un-denatured particle was also seen at ~70 kDa and similar observations are reported earlier^7^.

### Estimation of enzyme activity and stability in SLP

Enzyme activity of the lyophilized lipid particles encapsulated with L-asparaginase on day 1 and after 25 days of storage at 25 °C is as given in Table 1. Almost 100% activity was retained; the protein content and specific activity also remained the same.

**Table 1:** Estimation of L-asparaginase activity in SLP, stability, kinetics and pH studies.

Parameter/Condition tested	Enzyme preparation tested	
**Stability when stored at 25 °C**	**L-asp**	**LPE**
Enzyme activity Day 1 (IU/mL)	24.55 ± 1.12	30.15 ± 1.56
Enzyme activity Day 25 (IU/mL)	2.62 ± 0.55	29.47 ± 2.74
**Kinetics for natural substrate L-asparagine**	**L-asp**	**LPE**
V_*max*_ (µmole/mL/min)	7.79 ± 0.34	10.21 ± 1.43
K_*m*_ (mM)	0.070 ± 0.01	0.04 ± 0.001
**Optimum pH for activity**	**L-asp**	**LPE**
Enzyme activity at pH 8.5	6.12 ± 0.21	7.70 ± 0.38
Enzyme activity at pH 9	5.50 ± 0.24	8.15 ± 0.36
**Trypsin tolerance test (half life)**	**L-asp**	**LPE**
Time (mins)	50.19	120.56

### Kinetics of lipid particle encapsulated L-asparaginase for L-asparagine hydrolysis

L-asparaginase from *Bacillus tequilensis* PV9W and the same enzyme encapsulated in lipid particle were tested for enzyme velocity for different concentrations of L-asparagine. The non-regression analysis is depicted as a Michaelis Menten plot and as Lineweaver-Burk plot as seen in Supplementary Fig. S6.

The corresponding K_*m*_ and V_*max*_ are given as in Table 1. The lipid particle encapsulated L-asparaginase showed improved V_*max*_ compared to the native L-asparaginase. Moreover, K_*m*_ value for lipid particle encapsulated L-asparaginase was reduced to half that of the native L-asparaginase from *Bacillus tequilensis* PV9W. This decrease in K_*m*_ shows improved substrate specificity of lipid particle encapsulated L-asparaginase.

Further, the L-asparaginase from *Bacillus tequilensis* PV9W showed a half-life of ~50 min while LPE showed ~120 min half-life in presence of trypsin. Thus LPE showed improved stability compared to native L-asparaginase (Supplementary Fig. S6b).

Moreover, LPE showed pH tolerance up to pH 9 compared to the native L-asparaginase which could be stored at only until pH 8.5 (Supplementary Fig. S6c). The improved pH resistance is perhaps the advantage of encapsulation in lipid particle (LPE).

### Cytotoxic activity of LPE on human cervical cancer cells (HeLa)

HeLa cells treated with native L-asparaginase from *Bacillus tequilensis* PV9W at its IC_50_ value showed 50% cell survival after 24 h. However, the cells treated with LPE (containing IC_50_ equivalent concentration of the enzyme) showed complete cell mortality observed by floating of cells within 24 h. The LPC on the other hand did not show any cell death and was equivalent to the untreated HeLa cells (Supplementary Fig. S7).

Hence the MTT analysis was performed with lyophilized powders of L-asparaginase (native), LPC and LPE. 1 mg/mL each stock was made and the enzyme activity present in 1 mL was estimated by direct nesslerization. The stock volume of 1–10 µL was used to treat the HeLa cells and the resultant enzyme activity and the IC_50_ value on HeLa cells are as given in Table 2.

**Table 2:** Enzyme preparations and their IC_50_ values for HeLa cell cytotoxicity.

Treatment	Enzyme content	IC_50_ value
L-asp	40 IU/mg/mL	0.133 IU
LPC	0/mg/mL	No cytotoxicity
LPE	15 IU/mg/mL	0.085 IU

### L-asparaginase cytotoxicity and L-asparagine depletion

L-asparagine concentration in HeLa cells treated with L-asparaginase and the un-treated control were estimated by HPLC of the cell extracts. HPLC chromatograms of HeLa cell extracts of LPE treated and untreated conditions at initial 0 h of treatment and 8^th^ h after treatment are given by (Supplementary Fig. S8). The standard L-asparagine peak was seen at retention time (RT) of 5.1, which is similar to previous reports^10^ and it is seen as that of the HeLa cell extracts also.

### Differential Pulse Voltammetry (DPV) for measuring L-asparagine content in HeLa cells treated with L-asparaginase and LPE- A comparative study

**Figure 4:**
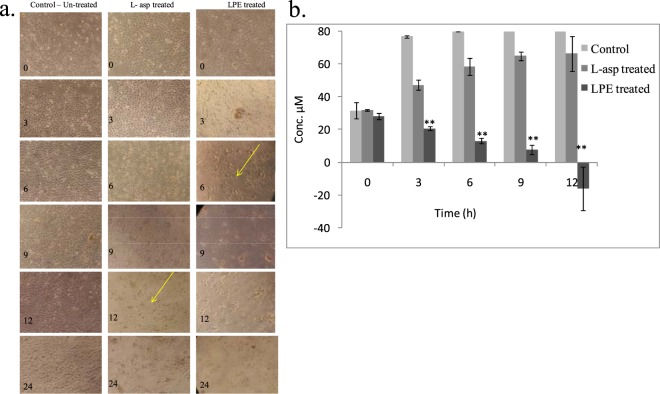
Analysis of L-asparagine depletion in HeLa cells treated with LPE. (**a**) Microscopic observation (phase contrast) of HeLa cells untreated, L-asparaginase treated and LPE treated at different hours after treatment. (**b**) L-asparagine content in HeLa cells in un-treated (control), L-asparaginase treated and LPE treated at different hours after treatment (LPE treatment showed significant difference over control in depletion of L-asparagine from 3^rd^ hour to 12^th^ (p < 0.01) (See Supplementary Plate 5).

Glassy carbon electrode (GCE) coated with LPE was used as a sensor to detect L-asparagine in an electrochemical cell by using a three electrode system (Supplementary Fig S9), Standard L-asparagine of different concentrations from 5 µM to 100 µM was applied to the cell and the voltammograms where L-asparagine showed a peak at −0.230 V. Similar detection was tested for L-glutamine in the electrochemical cell. However, L-glutamine did not show any oxidation peak with this electrode. Thus the specificity of the electrode to detect only L-asparagine is conserved. Cell extracts of HeLa which were treated with native L-asparaginase and with LPE along with the untreated cells were obtained at different hours of treatment. The delta I values obtained at −0.230 V for each sample was used to calculate the L-asparagine concentration using the graph obtained by standard L-asparagine (Supplementary Fig. S10). The L-asparagine content for each treatment and time was plotted as graph and depicted as Fig. 4. The graph denotes the depletion of L-asparagine content from 3 h to 12 h of treatment by LPE. L- asparagine is a vital amino acid exchange factor that regulates protein biosynthesis and hence the efficacy of clinical treatments of asparaginase in low-asparagine synthetase (ASNS)-expressing cancers have been explained^11^. However, in solid tumours the ASNS activity causes production of L-asparagine from L-glutamine as a substrate which is present in serum or supplied in cell culture media as in the present case. Thus, cancer cells may evade asparaginase sensitivity by up-regulating ASNS expression at times, in order to recover their intracellular asparagine pools by ASNS-catalysed synthesis from glutamine^11^. Thus, there is an increase in L-asparagine content in proliferating cancer cells and treatment of larger amount of L-asparaginase may be needed, or a longer time may be required by L-asparaginase alone to cause cell death. In the present study, L-asparaginase alone took nearly 12 h to cause cell death and depletion of L-asparagine was slower compared to LPE treatment. Thus treatment of HeLa cells by LPE in the present study demonstrates enhanced, depletion of L-asparagine resulting in faster cell death before a salvage mechanism is made by the ASNS expression by cancer cells.

Further, microscopic observation of the HeLa cells at various hours of treatment revealed that un-treated HeLa cells tend to remain viable and confluent till 36 h, while the cells treated with L-asparaginase shows gradual cell death after 12 h of treatment however, LPE treated cells show significant cell death after 6 h of LPE administration (Fig. 4).

### Effect of L-asparaginase from *Bacillus tequilensis* PV9W, recombinant L-asparaginase *pET28a- ansA*, and LPE on RAW 264.7 macrophage cell line

**Figure 5:**
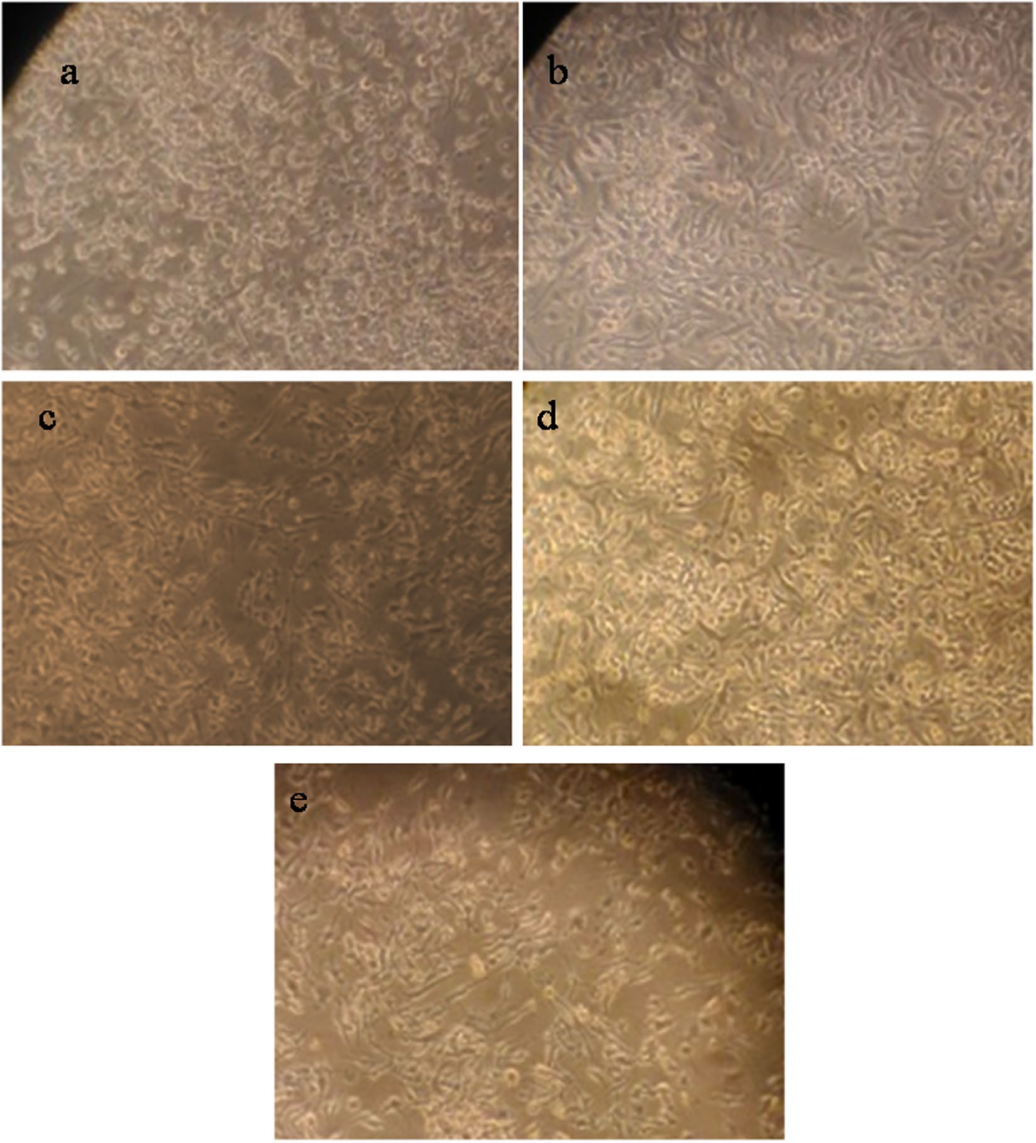
Microscopic observation (phase contrast) of RAW 264.7 macrophage cells. (**a**) Untreated control, (**b**) induced by LPS (+ve control), (**c**) L-asparaginase from *B. tequilensis* PV9W, (**d**) LPE (lipid particle encapsulated L-asparaginase from *B. tequilensis* PV9W), (**e**) L-asparaginase from *pET28a-ansA* clone.

All the three L-asparaginase preparations in this work were tested on RAW 264.7 primary cell lines and its effect was observed for formation of distinct dendritic morphology in case of inflammation as seen in positive control LPS induced^12^ (Fig. 5). Though L-asparaginase from *Bacillus tequilensis* PV9W and recombinant L-asparaginase *pET28a-ans*A, showed dendritic morphology, while LPE treated cells remained in its normal form.

## Discussion

Recombinant DNA technology has been undertaken as a strategy to over produce commercially important therapeutic enzymes like L-asparaginase in another bacterial host of different genus^13,14^. This enzyme is regulated by different genetic elements in different genus of bacteria. In *Bacillus*, the *ans*A gene is located in an operon along with *ans*B, which encodes L-aspartase and the expression of the *ans*AB operon is repressed by AnsR, and the activity of AnsR has been proposed to be regulated by either asparagine or aspartate^3^. The cloning, over-expression, and characterization of the gene encoding L-asparaginase (*ans*Z) from a non-pathogenic strain of *Bacillus subtilis* B11−06 has been reported. The protein was successfully purified and characterized for its thermo-stability^4^. The PCR based screening of *ans*A gene in *Bacillus tequilensis* has been reported by Nayak *et al*., (2014)^5^. This study involves isolation of the *ans*A gene and a first attempt in cloning it in suitable strain for over expression.

In this study, the *ans*A gene in *Bacillus tequilensis* PV9W was cloned into *E. coli* BL21 (DE3) using pET28a+ vector between the restriction sites for the enzymes *EcoRI* and *Hind III* at the multiple cloning site (MCS). Use of pET vector series have been reported for successful cloning of this enzyme before for expression of intracellular enzyme^14^. The clones were confirmed to have complete ORF for L-asparaginase biosynthesis. The over-expression of L-asparaginase in *E. coli* BL21 was achieved by 1 mM IPTG induction and purified by Ni-NTA column to homogeneity and verified by native and SDS-PAGE. The purified protein is His tagged at N terminal and the purification of the His tagged protein is confirmed by the immunoblot analysis using the anti-His antibody. The His tag is useful in protein purification by increased specificity and minimized loss as seen by the purification profile table and, it does not affect the structure of the protein in most of cases^15^. The purified protein is thus confirmed as the recombinant protein which was functionally active. The cloning and over-expression of L-asparaginase from *Bacillus tequilensis* PV9W in *E. coli* BL21 (DE3), to the best of our knowledge it is the first report for the *Bacillus tequilensis*. The enzyme yield obtained by molecular intervention of *ans*A gene in *E. coli* BL21 in 12 h cultivation showed yield similar to that of *Bacillus tequilensis* PV9W obtained in 48 h however retaining the same cytotoxic ability as in previous reports^1^. This prospect is of interest in commercial preparation of the L-asparaginase.

Further, to enhance the drug delivery and long lasting effect of L-asparaginase from *Bacillus tequilensis* PV9W was encapsulated into solid lipid particles synthesized using palmitic acid based method was reported previously for drug delivery of itraconazole^16^. Several methods have been so far used to improve the drug delivery and stability of L-asparaginase including immobilization by poly ethylene glycol (PEG)^17,18^ and many such advances that are reviewed^6^. Immobilization offers many advantages that can modify L-asparaginase to increase half-life and lower immune reactivity and have been reviewed extensively^19^. Liposomes and lipid particle have been explored for bio-compatible and efficient drug delivery. The disadvantages of liposomes have been overcome by lipid bio-conjugation and also the limitation of other lipidic structures like the insufficient loading capacity for most proteins with hydrophilic nature^7^. However the procedure involved the covalent linkage of fatty acids having different chain lengths (C_12_, C_16_ and C_22_) to the native enzyme. Alternatively solid lipid particle (SLP) synthesis involves a simplified procedure that offers advantage of incorporating both hydrophilic and hydrophobic interactions. SLP have been reported for itraconazole^16,20^. Hence, in this work, L-asparaginase was encapsulated in SLP and its characterization was studied to enhance its efficiency.

The particle nature of the lipid encapsulation (SLP + L-asparaginase) was verified by SEM analysis. The particle of size of nearly 100 nm and a small number of bigger size particle was similar to SLP loaded with primaquine^21^. The loading of the enzyme into the particle was confirmed by UV-Vis spectroscopy and FT-IR analysis where a H-OH bonding was seen between the enzyme and the particle. This result is in accordance with previous characterization reports^20,21^. The TGA and trypsin half-life analysis of L-asparaginase and lipid encapsulated L-asparaginase also are an evidence for improved stability to thermal biological stress of trypsin and pH.

Moreover, the particle showed enhanced enzyme activity, lowered K*m* increased stability in storage and doubled trypsin half-life compared to the native enzyme. Further, it had an improved cytotoxic action on HeLa cells in lesser IC_50_ value and within 12 h. Similar reports of increased cytotoxicity of lipid particle encapsulated drug was in accordance to the curcumin loaded polymer encapsulated in lipid particle^22^. The mode of action of L-asparaginase encapsulated particle (LPE) was confirmed by the depletion of L-asparagine in cells and was confirmed by HPLC analysis of cell extracts treated with lipid particle encapsulated L-asparaginase. HPLC was used to detect L-asparagine^10^, and reports of leukemic cells analyzed for L-asparagine levels by UPLC and HPLC^23^. However, in this work, HPLC chromatograms of HeLa cell extracts treated with LPE showed many non specific peaks in similar retention time. Hence it was not specific to L-asparagine and specificity was not ascertained as there may be many ureides which show a similar RT^10^.

Therefore another sensitive method involving electrochemistry using differential pulse voltammetery (DPV) was employed using LPE coated glassy carbon electrode (GCE) to specifically detect L-asparagine without L-glutamine interference. The results were promising and the LPE were more effective in L-asparagine depletion than plain L-asparaginase alone at a statistically significant rate (Supplementary Plate 5). This might perhaps be an outcome of improved efficiency of the LPE due to increased substrate specificity (K_*m*_) and enhanced thermal and biological stability. The cytotoxic effect was highly specific and fast acting in HeLa cell lines. Thus LPE can be prospected as an efficient therapeutic agent with an prolonged storage, stability and cytotoxic potentials.

Furthermore, all the three enzyme preparations in this work, namely, L-asparaginase from *Bacillus tequilensis* PV9W, recombinant L-asparaginase from *pET 28a- ans*A clone1, and LPE were tested for inflammatory response on RAW 264.7 macrophage cell line and LPE treated cells did not show any immunological response of inflammation when tested *in vitro*. RAW264.7 cells are mature macrophages and their differentiation into dendritic cells as a mechanism where tissue macrophages are recruited to become dendritic cells during infection, a conversion that would be beneficial for mounting an effective immune response^12^. Here, this conversion to dendritic form by macrophage cells is an indication of such inflammatory response. However, LPE treatment did not elicit any immune response and hence LPE can be a remarkable candidate for therapeutic regimen involved in anti-cancer therapy.

## Conclusion

The *ans*-A gene coding for L-asparaginase from *Bacillus tequilensis* PV9W was cloned and over expressed in a heterologus host for the first time, which revealed an equivalent production of L-asparaginase could be achieved in 12 h compared to the native enzyme in 48 h. Moreover, lipid particle encapsulated L-asparaginase revealed an improved stability in storage, pH and trypsin tolerance and higher thermo stability. Further, an enhanced cytotoxicity in relatively less time was achieved and demonstrated by electrochemical methods. This LPE preparation was also used to assess L-asparagine concentration in cell extracts paving a way for bio sensing of the amino acid which could have a future scope in cancer diagnosis.

## Methods

### PCR amplification of *ans*A gene from *Bacillus tequilensis* PV9W and its expression in *E. coli* BL21 (DE3)

Genomic DNA from *Bacillus tequilensis* PV9W (GenBank Accession number: KR261609) was extracted by a non enzymatic method^24^ and *ans*A gene was amplified using the ansA ORF primers designed using the ORF sequences of *ansA* region of *Bacillus tequilensis strain* NIOS4 L-asparaginase I (*ans*A) gene, complete cds (Gen Bank -JQ911764.1).

The eluted *ans*A was ligated into pGEM-T vector, transformed into competent *E. coli* DH5α and plated onto LB agar plate containing ampicillin. The plasmids from the positive clones selected by blue white selection were further subjected to restriction digestion using *EcoR*I and *Hind* III. The released insert from the confirmed clone was further re-ligated into pET *28a*+ vector and transformed into competent cells of *E. coli* BL21 (DE3) and then selected by kanamycin (Himedia, India) resistance.

The recombinant colonies on kanamycin plates were screened through colony PCR using forward primers specific for the *ans*A gene and restriction digestion analysis of the plasmids of the clones was performed with the enzymes *Eco R*I and *Hind* III. Further, the sequence analysis of the isolated plasmid pET 28a+ -*ans*A was obtained to confirm presence of *ans*A gene.

Recombinant cells were grown in LB medium (5 mL) for 6 h and induced with different concentration of IPTG (Himedia, India) (0.5 mM, 1 mM) at 25 °C for 3 h. The cells were separated by centrifugation washed twice with PBS buffer (pH 7.4), suspended in 50 mM Tris-HCl (Himedia, India) buffer (pH 7.5) and subjected to sonication. Cell extracts were obtained by soniction of cells in 50 mM Tris-HCl buffer and centrifugation at 5 min at 10,000 g to remove cell debris. The supernatant was used to analyze by gel electrophoresis (SDS-PAGE) analysis or were stored at −20 °C until further analysis. The *E. coli* BL21 (DE3) transformed with pET 28a+ without *ans*A insert was used as a negative control.

### Purification of recombinant L-asparaginase produced by pET 28a+ -*ans*A clone

L-asparaginase (N-terminal His tagged) was over expressed in pET 28a+ -*ans*A clone which was grown in LB medium (200 mL) for 6 h initially followed by IPTG induction for 3 h at 25 °C which was standardized by many previous experiments. Initial experiments involved 1 h, 2 h, 3 h and 5 h induction but for 5 h induction the cell lysate had less enzyme activity than the 3 h. Hence 3 hr induction was standardized. The cell free extract of sonicated bacterial cells was the crude extract and was purified on a Ni-NTA column using His-Tagged Bacterial Protein Purification Kit (Spin Column), Hi-Media, India. The purified protein was quantified by direct nesslerization method^25^ and the yield was compared to the crude protein. The western blot analysis of the purified protein was performed to verify the expression vector using anti-His primary antibody (Sigma, Cat no. H1029; Lot-121M4789) followed by HRP conjugated secondary antibody against the primary antibody. The molecular weight of the protein bands was calculated by comparison with SDS-PAGE marker (Gene direx, Taiwan).

### Functional activity of purified recombinant L-asparaginase produced by *E. coli* pET 28a-*ans*A clone by M-9 plate assay

The production of functional L-asparaginase by IPTG induced pET 28a-*ans*A clone was verified compared to the control BL21 strain and the un-induced clone, by plating the culture on M-9 plate containing L-asparagine as sole nitrogen source, D-glucose (0.3% w/v) and phenol red indicator. The plates were observed after 16 h for growth and colour development.

### Cytotoxic activity of L-asparaginase produced by *E. coli* pET 28a-*ans*A clone on human cervical cancer cells (HeLa)

HeLa cells were cultured in DMEM (high glucose) (Himedia, India) media supplemented with 10% FBS (Hi-Media, India) and 1% antibiotic penicillin/streptomycin (Hi-Media, India) mixture and seeded on 96-well plates (1 × 10^4^ cells/well) and incubated at 37 °C in a 5% CO_2,_ humidified atmosphere. After attaining confluence (12 h), the recombinant L-asparaginase purified from, *E. coli* pET 28a-*ans*A clone was given at different concentrations to determine its cytotoxicity compared to the untreated control HeLa cells.

### Bio compatible carrier particle for drug delivery Solid Lipid Particle (SLP)

#### Preparation of SLP

SLPs were prepared by the melt-emulsion sonication and low temperature-solidification method^15^. Briefly, 500 µL (25 IU/3 mg weight lyophilized purified enzyme) of L-asparaginase from *Bacillus tequilensis* PV9W, dissolved in buffer, was mixed with 450 mg of lipid and the mixture was heated up to 70 °C. This emulsion of the lipid drug solution was added drop-wise to 10 mL of PVA (4%) solution (70 °C) and further subjected to sonication for 10 mins. This emulsion was quickly poured into chilled water (20 mL) under constant stirring. The stirring was continued until the emulsion yielded a uniform dispersion of particles. The particles were separated by centrifugation (15,000 rpm, 4 °C) for 5 min., lyophilized and re-suspended in milli-Q (MQ) water and stored in −80 °C.

### Characterization of SLP

The characterization of the lipid particles was done by UV-visible absorption spectroscopy where the solid lipid particles containing the L-asparaginase (LPE), control lipid particle (LPC) without enzyme, and purified L-asparaginase from *Bacillus tequilensis* PV9W were analyzed individually. Further, their FT-IR spectra were obtained (Shimadzu, Japan). SEM analysis (VEGA3 TESCAN) was performed for the LPE sample to observe its surface morphology observed at 5.00 kx magnification. PAGE analysis of the particle encapsulating the L-asparaginase was performed under denaturing conditions (SDS) to ensure the L-asparaginase loading into the particles^7^. The purified L-asparaginase was also loaded onto the gel to serve as control.

The solid lipid particles containing the L-asparaginase were assayed for their enzyme activity by Mashburn Wriston method of direct nesslerization^25^. The enzyme activity was also calculated after 25 days of storage in phosphate buffer at 25 °C.

LPE and purified L-asparaginase from *Bacillus tequilensis* PV9W was assayed for enzyme activity by direct nesslerization for different concentrations of L-asparagine. The non-regression analysis of the activity vs. substrate conc. was plotted as a Michaelis Menton as well as Lineweaver Burk plot.

Thermogravimetric analysis (TGA) was used to measure the physical and chemical changes of the nanoparticles as a function of temperature. Lipid particle encapsulated enzyme (LPE), Lipid particle control (LPC) and L-asparaginase alone was analyzed using TGA (TGA-601; PerkinElmer Inc., Waltham, MA, USA) connected to an inert nitrogen gas flow and at a heating rate of 10 °C/min^21^.

Stability in trypsin for L-asparaginase and LPE were tested in presence of trypsin^26^. Further, optimum pH for the enzyme activity was analyzed as performed for enzyme characterization previously.

### Cytotoxic activity of LPE on human cervical cancer cells (HeLa)

HeLa cells were cultured in DMEM (high glucose) (Hi-Media, India) media as per standard procedures mentioned before. After attaining confluence, the cells were treated with different concentrations of native L-asparaginase purified from *Bacillus tequilensis* PV9W at its IC_50_ value, Lipid particle control (LPC) and Lipid particle encapsulated enzyme (LPE), (prepared from 1 µg/mL stock of each lyophilized preparation). MTT was performed and the IC_50_ value was calculated in µL of stock utilized followed by the calculation of corresponding enzyme activity. This IC_50_ value of LPE and L-asparaginase was used for all further assays.

### Mode of action of LPE cytotoxicity on HeLa cells – L-asparagine depletion assay methods

#### L-asparagine content in LPE treated HeLa cells by HPLC analysis

In order to ensure that the activity of LPE is by effective depletion of L-asparagine in HeLa cells and to compare its efficiency with native L-asparaginase, the HeLa cells were cultured in a 6 well plate at cell density of 1 × 10^6^ cells and given native enzyme and LPE at IC_50_ value (in triplicate). After 12 h incubation, all the cells were harvested, washed with phosphate-buffered saline (PBS), dried and processed as done for UPLC-TOF analysis in an earlier report^23^. After precipitation of the protein content, the supernatant was evaporated, and the dried pellets were re-suspended in 150 μL of water/acetonitrile/formic acid (39.9/60.0/0.1 v/v/v %) and centrifuged. The supernatant thus obtained was tested by HPLC for L-asparagine content on C18 column, 5 μm, 250 mm × 4.6 mm, mobile phase was acetonitrile: 0.03 M potassium phosphate, pH 3.2 (20:80). The flow rate was 0.5 mL/min at a column temperature of 30 °C and sample injection volume was 20 μL. A different range of concentration of L-asparagine (10–100 μmoles mL^−1^) made in acetonitrile: 0.03 M monobasic potassium phosphate pH 3.2, (20:80) was used as standard and the peaks were detected at 190 nm^10^. The corresponding peak obtained at same retention time (RT) in cell extract as the standard L-asparagine was considered as L-asparagine and its concentration was calculated by area of the peak in the HPLC study that was performed in three independent repeats.

### L-asparagine content in LPE treated HeLa cells–Differential pulse voltammetry (DPV) method

DPV was used to measure minute concentration differences of L-asparagine (20 μM to 110 μM) using glassy carbon electrode GCE coated with Lipid particle containing L-asparaginase (LPE) bound on electrode with 0.1% Nafion (Sigma, USA) resin. This LPE coated GCE is used to sense the increasing concentration of L-asparagine. The potential between the working electrode (GCE with LPE) and the reference electrode (Ag-AgCl_2_) is changed as a pulse from an initial potential (−1) to an inter level potential and remains at the intermediate potential for about 100 milliseconds; then it changes to the final potential (+1). The pulse is repeated, changing the final potential, and a constant difference is kept between the initial and the inter level potential (pulse height P_H_ = 50.0 mV, Pulse width P_W_ = 50.0 ms, Step height S_H_ = 5.0 mV, step time S_T_ = 500.0 ms, Current range I = 10 mA). The value of the current between the working electrode and auxiliary electrode before and after the pulse are sampled and their differences are plotted versus potential. These measurements can be used to study the redox properties of extremely small amounts of L-asparagine that binds specifically to the L-asp in LPE coated on electrode. Similarly, L-glutamine is also tested to verify for cross-reactivity.

### Preparation of extracts from HeLa cells

HeLa cells were cultured in a 6 well plate at cell density of 1 × 10^6^ cells and given native enzyme and LPE both at their respective IC_50_ value (in triplicate). After 12 h incubation, all the cells were harvested, washed with PBS and dried. The cells were processed as done for HPLC analysis before^23^, however after precipitation of the protein content, the supernatant was evaporated, and the dried pellets were re-suspended in 1 mL of PBS.

The LPE treated and L–asparaginase treated HeLa cell extracts obtained above after 0, 1, 3, 6, 9, 12 and 15 h of incubation were tested in three electrode system described above and the redox peak was documented as current versus potential graph. The concentration of L-asparagine in test extract is calculated at the −0.230 V using standard L-asparagine graph.

The morphological observation of the cells treated with L-asparaginase and LPE were also made using phase contrast microscopy and documented for 0, 1, 3, 6, 9, 12 and 15 h of incubation after cells were treated. In both cases, untreated HeLa cells served as control.

### Effect of L-asparaginase on RAW 264.7 macrophage cell line

The RAW 264.7 cells (1 × 10^6^ cells/well) were seeded in 10 mm diameter petri dishes, cultured in DMEM with 10% fetal bovine serum. After the cells attained confluence, they were treated individually with native L-asparaginase, recombinant L-asparaginase and LPE and incubated in CO_2_ incubator. Untreated and LPS treated (1 µg/mL) cells were kept as control. The morphological changes in the cells were observed microscopically up to 24 h of growth.

## Electronic supplementary material

Supplementary Information
