# Supplementary material for: Cloning and expression of L-asparaginase from Bacillus tequilensis PV9W and therapeutic efficacy of Solid Lipid Particle formulations against cancer
Source: Sci Rep. 2018 Dec 20;8:18013. doi: 10.1038/s41598-018-36161-1 (PMC6301963; doi:10.1038/s41598-018-36161-1)
Supplement: Supplementary file 1 — Supplementary Information [file 41598_2018_36161_MOESM1_ESM.doc]

**Cloning and expression of L-asparaginase from *Bacillus tequilensis* PV9W and therapeutic efficacy of Solid Lipid Particle formulations against cancer**

**Ganeshan Shakambari^1^, Rai Sameer Kumar^1^, Ashokkumar Balasubramaniem^2^, Venkatachalam Ganesh^3^,** **Vairathevar Sivasamy Vasantha^4^, Perumal Varalakshmi^1*^**

*^1^Department of Molecular Microbiology, School of Biotechnology, Madurai Kamaraj University, Madurai, Tamil Nadu, India, 625021*.

*^2^Department of Genetic Engineering, School of Biotechnology, Madurai Kamaraj University, Madurai, Tamil Nadu, India, 625021*.

*^3^Electrodics and Electrocatalysis (EEC) Division, CSIR - Central Electrochemical Research Institute, (CSIR - CECRI), Karaikudi Tamilnadu, India, 630003.*

*^4^Department of Natural Products Chemistry, School of Chemistry, Madurai Kamaraj University, Madurai, Tamil Nadu, India, 625021*.

*Corresponding Author: Dr. Perumal Varalakshmi*.*

**Supplementary Fig. S1 a.** PCR screening *ans*A gene in *Bacillus tequilensis* PV9W, amplicon of about 990 bp . **b.** BLAST analysis of the sequences of the 990 bp amplicon obtained from *Bacillus tequilensis* PV9W. **c.** The sequence showed 99 % identity with L-asparaginase super family.


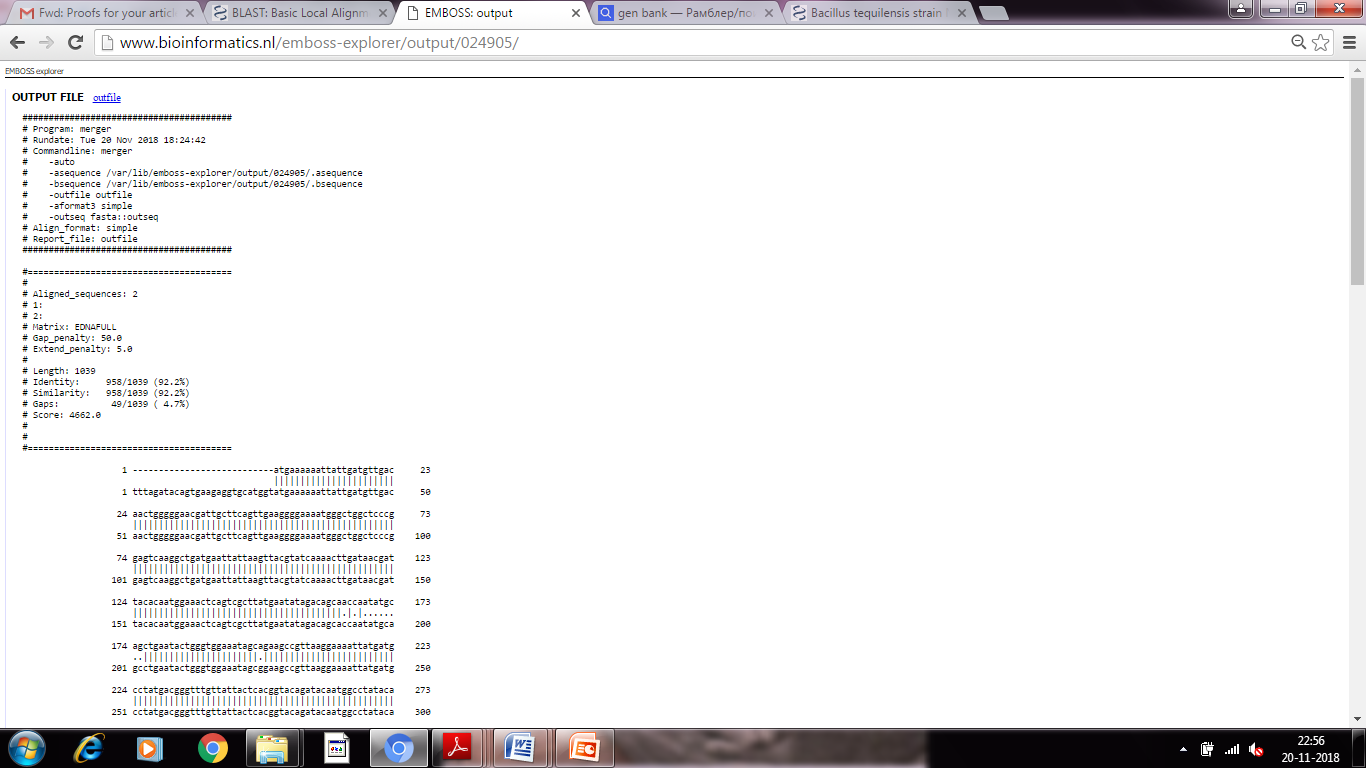


**Supplementary Fig. S2** EMBOSS output: Sequence alignment of *pGEMT - ansA* (clone 5) with complete cds from L-asparaginase I (*ans*A) gene, *Bacillus tequilensis strain* NIOS4 (JQ911764.1**)** .

**Supplementary Fig. S3 a.** Colony PCR analysis of four colonies obtained on *Kanamycin* plates, amplified using primers specific for *ans*A gene. Colonies 1, 2 and 4 were positive for the *ans*A gene (990 bp). L- 100bp ladder, 1, 2, 3 and 4 were PCR products from colonies 1, 2, 3 and 4. BL21 – Control *pET 28a+* (without insert). **b.** Restriction Digestion of 3 clones: *pET 28a- ans*A (clone 1, 2 and 4) by *EcoRI* and *Hind* III*.* L-1kb ladder. Lane 1, 2 - un-digested and digested *pET 28a- ansA* (clone 1), Lane 3, 4 - digested and un- digested *pET 28a- ans*A (clone 2), lane 5, 6 - digested and un- digested *pET 28a- ans*A (clone 4).

**Supplementary Fig. S4 a.** Sequence alignment of the *pET 28a- ansA* clone 1 revealed identity with *ans*A gene from *Bacillus tequilensis* strain NIOS4 L-asparaginase I (ansA) having entire ORF. **b.** The sequence obtained by Sangers di-deoxy sequencing of the *pET 28a- ans*A clone 1 corresponding the ORF is presented here along with the translated amino acid sequence

**Supplementary Fig. S5** Electrophoretic analysis of recombinant L-asparaginase cloned and over-expressed in *E.coli* BL21 (DE3) and purified using Ni NTA chromatography.
**a**. Native PAGE, **b**. SDS PAGE. L- Ladder, C- Crude cell lysate, P-Purified protein, F- Column Flow through, W- Column Washing. **c.** Microscopic observation of HeLa cells treated with recombinant L-asparaginase produced by *pET 28a- ans*A clone 1

**Supplementary Fig. S6 a.** Enzyme kinetics given by a. Michaelis–Menten plot of reaction velocity (V) vs. substrate conc. (S) for L-asparaginase and Lipid particle encapsulated L-asparaginase. **b.** Enzyme kinetics depicted by corresponding Lineweaver–Burk plot. **b. .** *In vitro* half-life of L-asparaginase in trypsin for L-asparaginase from *Bacillus tequilensis* PV9W and LPE. **c.** Effect of pH on enzyme activity of LPE and L-asparaginase.


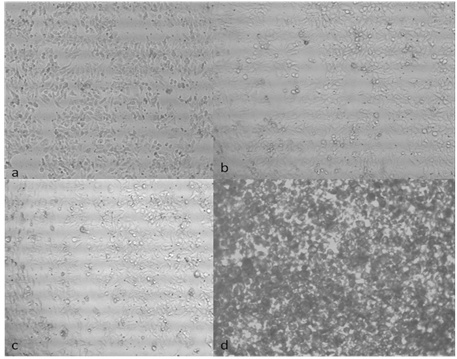


**Supplementary Fig. S7** Microscopic observation of HeLa cells. 24 h results **a**. Untreated HeLa cells. **b**. LP Control treated HeLa cells. **c**. L-asparaginase treated HeLa cells. **d**. LPE treated HeLa cells.


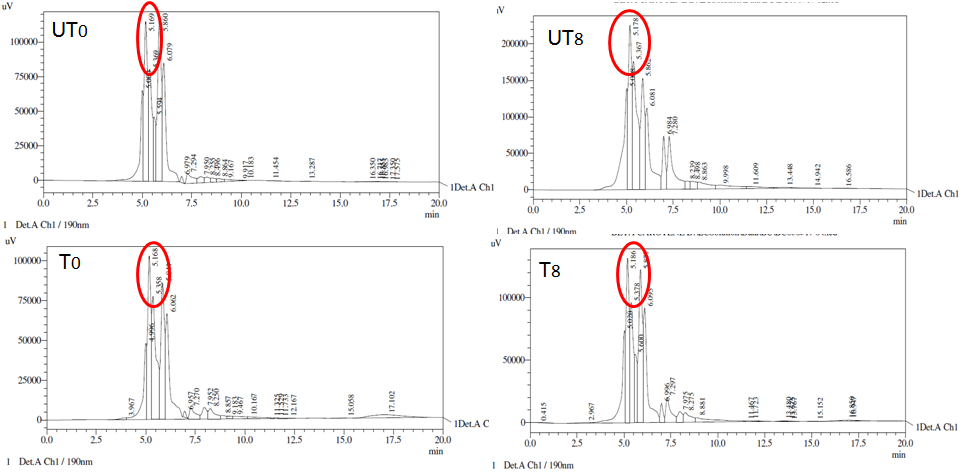


**Supplementary Fig. S8** HPLC chromatogram of cell extracts of HeLa cells **a.** un-treated (UT) and **b**.LPE treated (T) at 0^th^ h and 8^th^ h.

**Supplementary Fig. S9** DPV components **a.** GCE coated with LPE, **b.** Three electrode system.

**Supplementary Fig. S10 .** DPV plots for L-asparagine present in cell extracts of un-treated HeLa cells (C) and treated with L-asparaginase (A) and LPE (P) at different hours of treatment

**Supplementary Plate**

**
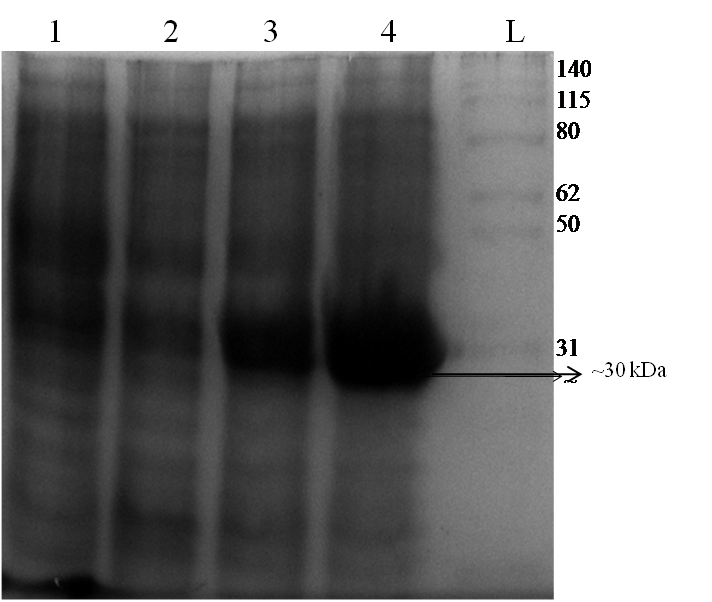
**

**Plate 1-** SDS PAGE analysis of recombinant L-asparaginase produced by *pET 28a- ans*A (clone 1) performed for confirming IPTG induced over expression of L-asparaginase . L- Ladder; cell lysates of 1- *E.coli* BL21–control; 2- pET clone 1 uninduced; 3- *pET 28a- ans*A (clone 1) induced with 0.5 mM IPTG; 4- *pET 28a- ans*A (clone 1) induced with 1 mM IPTG.


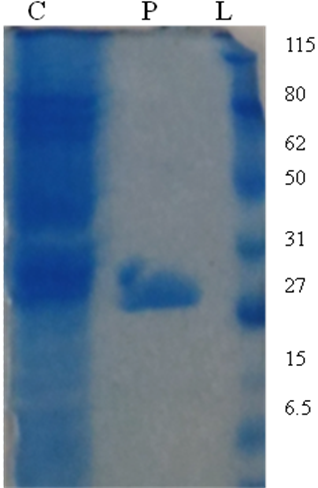


**Plate 2** SDS PAGE analysis, C-Crude enzyme from cell lysate, P- Ni-NTA column purified his-tagged L-asparaginase


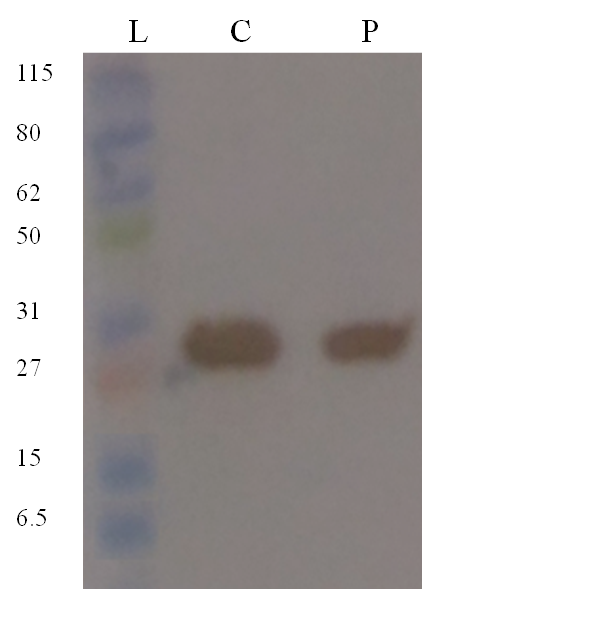


**Plate 3** Immunoblot of the membrane treated with anti-His antibody was developed with DAB ; L- Ladder; C- Crude cell lysate; P-Purified protein.


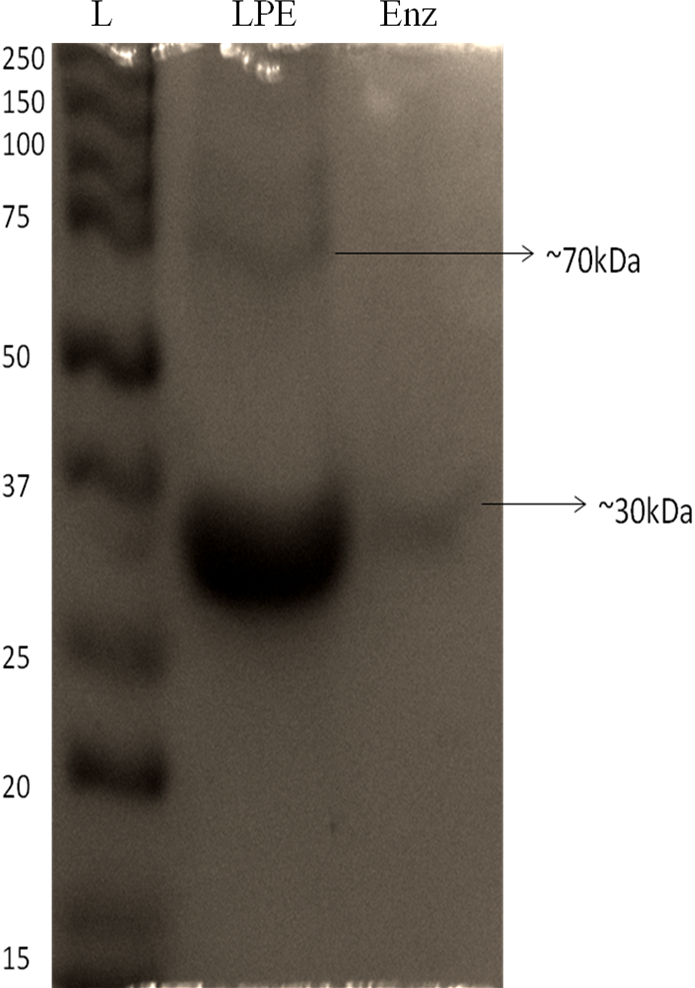


**Plate 4** SDS PAGE analysis of L-asparaginase and lipid particle encapsulated L-asparaginase and purified L-asparaginase from *Bacillus tequilensis* PV9W

**Plate 5:** L-asparagine content in HeLa cells in un-treated (control), L-asparaginase treated and LPE treated at different hours after treatment.

|  | L- asparagine content in extracts of HeLa cells with different treatments | | | ANOVA summary | | *Tukey HSD Test* |
| --- | --- | --- | --- | --- | --- | --- |
| Hour of treatment (h) | Untreated (Control)-**C** | L-asparaginase treated (**A**) | LPE treated (**LPE**) | F | P |  |
| 0 | 31.67 ± 3.86 | 28.15 ± 3.72 | 24.80 ± 4.3.59 | 0.85 | 0.47 | Not significant |
| 3 | 76.87 ± 0.44 | 47.25 ± 2.32 | 20.74 ± 1.03 | 355.56 | < 0.0001 | **C** *vs.* ***A*** p <0.01  **C** *vs****. LPE*** p<0.01  **A** *vs.* **LPE** p<0.01 |
| 6 | 80.54 ± 0.82 | 58.46 ± 5.19 | 13.16 ± 1.53 | 199.26 | < 0.0001 | **C** *vs.* ***A*** p <0.01  **C** *vs****. LPE*** p<0.01  **A** *vs.* **LPE** p<0.01 |
| 9 | 83.16 ± 1.10 | 65.04 ± 1.95 | 7.70 ± 2.28 | 454.45 | < 0.0001 | **C** *vs.* ***A*** p <0.01  **C** *vs****. LPE*** p<0.01  **A** *vs.* **LPE** p<0.01 |
| 12 | 85.06 ± 1.35 | 66.42 ± 7.94 | -15.87 ± 9.92 | 52.98 | 0.000154 | **C** *vs.* ***A*** not significant  **C** *vs****. LPE*** p<0.01  **A** *vs.* **LPE** p<0.01 |

* HSD = the absolute difference between any two sample means required for significance at the designated level (0.01). (LPE treatment showed significant difference over control in depletion of L-asparagine from 3^rd^ hour to 12^th^ (p< 0.01))
